# Supplementary material for: An antisense peptide-conjugated peptide nucleic acid (PPNA) for peptidoglycan recycling inhibition reduces AmpC hyperproduction and β–lactam resistance in Pseudomonas aeruginosa
Source: Microbiol Spectr. 2025 Jul 30;13(9):e02622-24. doi: 10.1128/spectrum.02622-24 (PMC12403870; doi:10.1128/spectrum.02622-24)

**Supplementary material 1.**

Certificate of Analysis of the PPNAs used in this study, provided by the manufacturer (PNA Bio Inc. Thousand Oaks, CA, USA).


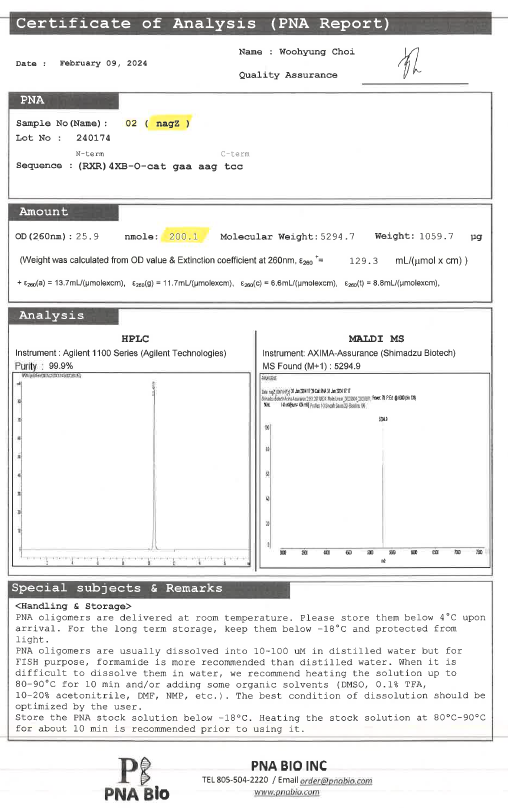


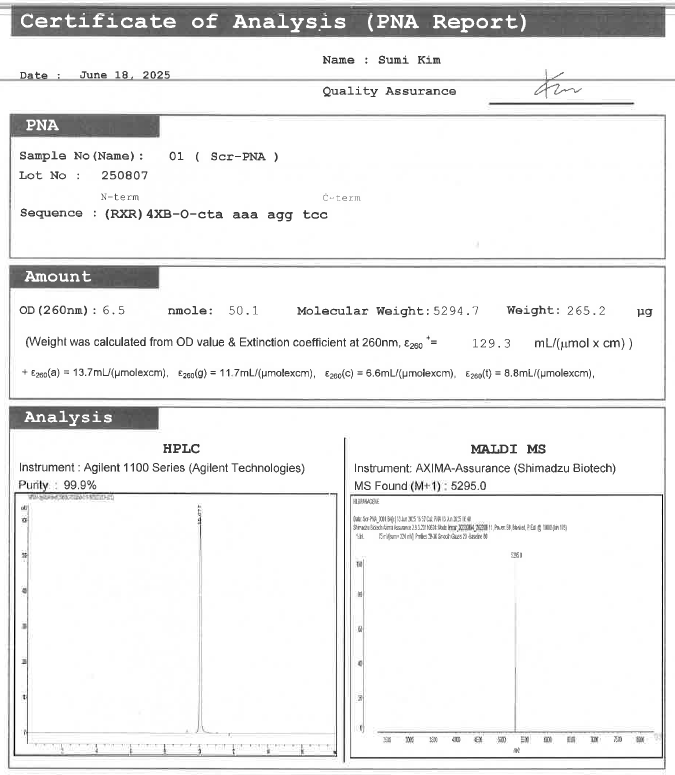


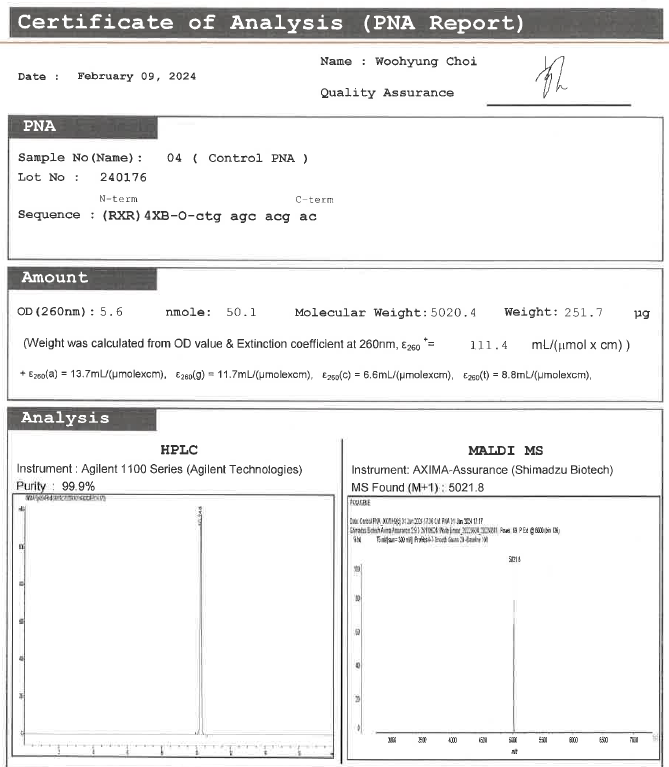

Supplement: Supplemental material — Certificate of analysis of the PPNAs used in this study, provided by the manufacturer. [file spectrum.02622-24-s0001.docx]
